# GABRD promotes progression and predicts poor prognosis in colorectal cancer

**DOI:** 10.1515/med-2020-0128

**Published:** 2020-11-21

**Authors:** Gengming Niu, Li Deng, Xiaotian Zhang, Zhiqing Hu, Shanliang Han, Ke Xu, Runqi Hong, He Meng, Chongwei Ke

**Affiliations:** Department of General Surgery, the Fifth People’s Hospital of Shanghai, Fudan University, 801 Heqing Road, Shanghai, 200240, People’s Republic of China; Department of Animal Science, School of Agriculture and Biology, Shanghai Jiao Tong University, Shanghai, 200240, People’s Republic of China

**Keywords:** GABRD, colorectal cancer, prognosis, growth, migration

## Abstract

Little is known about the functional roles of gamma-aminobutyric acid type A receptor subunit delta (GABRD) in colorectal cancer (CRC). The expression of GABRD between CRCs and adjacent normal tissues (NTs), metastasis and primary tumors was compared using public transcriptomic datasets. A tissue microarray and immunohistochemical staining (IHC) were used to determine the clinical and prognostic significance of the GABRD in CRC. We used gain-of-function and loss-of-function experiments to investigate the in vitro roles of GABRD in cultured CRC cells. We characterized the potential mechanism of GABRD’s activities in CRC using a Gene Set Enrichment Analysis (GSEA) with The Cancer Genome Atlas Colon Adenocarcinoma (TCGA-COAD) dataset. We found that the GABRD expression was significantly increased in CRCs compared to that in NTs, but was similar between metastasis and primary tumors. Overexpression of GABRD was significantly associated with later pTNM stages and unfavorable patient survival. Overexpression of GABRD accelerated while knock-down of GABRD inhibited cell growth and migration. Mechanistically, the function of GABRD might be ascribed to its influence on major oncogenic events such as epithelial–mesenchymal transition (EMT), angiogenesis, and hedgehog signaling. Collectively, GABRD could be a novel prognostic predictor for CRC that deserves further investigation.

## 1 Introduction

Colorectal cancer (CRC) is one of the most common malignancies in both east and west societies [1,2]. Despite recent progress in early screening and treatment [3], it remains a great challenge due to disease recurrence, drug resistance, and distal metastasis [4]. Identification of prognostic factors for CRCs may help select patients at a higher risk and develop more personalized therapies.

Gamma-aminobutyric acid (GABA) is the predominant inhibitory chemical neurotransmitter in the central nervous system. Originally, three groups of GABA receptors, namely GABA_A_, GABA_B_, and GABA_C_, were identified. The term GABA_C_ is no longer in use and now it is recognized that GABA_A_ is more prevalent and functionally related to GABA. GABA_A_ receptors are heteropentamers formed by five types of subunits, with a central chloride ion-selective channel gated by GABA [5,6]. Gamma-aminobutyric acid type A receptor subunit delta (GABRD), which encodes the GABA_A_ receptor δ subunit, has been suggested as a susceptibility gene to childhood-onset mood disorders and generalized epilepsies [7,8]. Several recent studies have revealed the possible functional roles of GABRD in tumors. In a cohort of patients with corticotroph adenomas, Bujko et al. demonstrated that GABA-related genes including GABRD were enriched in tumors with USP8 mutations, which are driver mutations in corticotrophinomas [9]. Using data from The Cancer Genome Atlas (TCGA), Gross et al. conducted a pan-cancer analysis and found that GABRD was overexpressed in nearly 90% of the patients included [10]. In another TCGA-based bioinformatic study, GABRD expression was significantly decreased in IDH wild-type diffuse low-grade gliomas compared with that in IDH mutant tumors, while patients with a high expression of GABRD had better prognosis than those with a low expression of GABRD [11]. Although Fagerberg et al. carried out a human tissue-specific expression analysis and demonstrated that GABRD mRNA was relatively abundant in the colon under physiological conditions [12], little is known about the involvement of GABRD in tumorigenesis and progression of CRCs.

In the present study, we analyzed the GABRD expression in CRCs and peritumoral normal tissues (NTs) with transcriptomic datasets from gene expression omnibus (GEO) and the cancer genome atlas (TCGA). We used clinically resected samples to validate these results with quantitative polymerase chain reaction (q-PCR). We also compared the expression levels of GABRD between primary and metastatic CRCs. We investigated the correlation between GABRD expression and patient survival with a tissue microarray by immunohistochemistry (IHC) and validated the result with the combined TCGA-colon adenocarcinoma (COAD) and rectal adenocarcinoma (READ) dataset. We investigated the in vitro roles of GABRD on cell proliferation and migration in cultured CRC cells using gain-of-function and loss-of-function assays. We characterized the possible mechanisms of GABRD’s function in CRC carcinogenesis using a gene set enrichment analysis (GSEA) with the TCGA-COAD dataset. We aimed to evaluate the prognostic value and oncogenic roles of GABRD in CRCs.

## 2 Patients and methods

### 2.1 Human CRC samples and cell lines

This study was approved by the Shanghai Fifth People’s Hospital Institutional Ethics Committee (Ethical Approval Form Number: 2017-097) and adhered to the principles listed in the Declaration of Helsinki. Informed consent was obtained before the collection of tissues. Sixteen paired tumor and NTs were collected from CRC patients at the Shanghai Fifth People’s Hospital (Shanghai, China) between 2016 and 2018. The samples were snap-frozen in liquid nitrogen and stored at −80°C. The corresponding formalin-fixed and paraffin-embedded tissues were retrieved, and 4-µm tissue sections were prepared by the Department of Pathology at the same hospital. Tissue microarrays were prepared by Shanghai Outdo Biotech (Shanghai, China) and contained CRCs from 100 patients with a median follow-up of 30 months. Detailed information about these samples is summarized in Table S1.

Five human CRC cell lines COLO205, COLO320DM, HCT116, HT15, and HT29, as well as a colon epithelial cell line FHC were obtained from the Cell Bank of the Chinese Academy of Sciences (Shanghai, China). Cells were cultured in McCoy’s 5A or DMEM supplemented with 10% FBS, 100 µg/mL of penicillin, and 100 mg/mL of streptomycin at 37°C with 5% CO_2_ in a humidified incubator (Thermo Fisher Scientific, Waltham, MA).

### 2.2 IHC

Sections were stained with a polyclonal antibody against GABRD (1:50 dilution; rabbit anti-human, mouse, and rat; abs141150; Absin Bioscience Inc., Shanghai, China) by IHC based on a standard protocol in the Department of Pathology at our institution [13]. Briefly, formalin-fixed and paraffin-embedded microarray sections were deparaffinized, dehydrated, and immersed in sodium citrate buffer (pH 6.0) for antigen retrieval, blocked with 3% hydrogen peroxide for 10 min at room temperature to inactivate endogenous peroxidase, rinsed with phosphate-buffered saline for 10 min, and pretreated in a microwave oven for 10 min. Then the slides were incubated at 4°C overnight with the primary antibody. After staining with a two-step plus Poly-HRP anti-Rabbit IgG Detection System (Elabscience Biotechnology Co. Ltd, Wuhan, China), the slides were visualized by reacting with the DAB chromogen (Biocare Medical LLC, Pacheco, CA, USA) counterstained with hematoxylin and covered with glycerin gel, and then photographed under a microscope. A modified H score system was used to semi-quantify GABRD expression, as previously described [14]. Briefly, the maximal intensity of staining (0, negative; 1, weak; 2, moderate; and 3, strong) was multiplied by the percentage of positive tumor cells (0–100%) to generate the modified H score (range: 0–300). The GABRD expression was classified into high or low by the median H score. Data interpretations were made independently by two pathologists who had been blinded to each other’s findings and to the original pathology reports.

### 2.3 Access to public datasets

We comprehensively searched transcriptomic datasets of CRCs in the GEO database [15]. Only those that compared gene transcription between NTs and CRCs, or between primary and metastatic CRCs were selected and further screened. Datasets that contained less than 50 tissue samples or provided incomplete information were ruled out. For repetitive datasets, after each data matrix was checked and compared with regard to sample ID and data type, only the one with most samples was kept. Ten datasets were finally included to compare the GABRD expression between NTs and CRCs, including GSE3629 [16], GSE6988 [17], GSE21510 [18], GSE28000 [19], GSE31279 [20], GSE37182 [21], GSE41258 [22], GSE44861 [23], GSE87221 [24], and GSE106582 [25]. In addition, the Colon Adenocarcinoma and Rectum Adenocarcinoma Projects of The Cancer Genome Atlas (TCGA-COAD and READ) were retrieved from UCSC Xena (https://xenabrowser.net/heatmap/) and combined into one CRC dataset.1The results <published or shown> here are in whole or part based upon data generated by the TCGA Research Network: http://cancergenome.nih.gov/
 These 11 datasets include 707 NTs and 1358 CRCs. Besides, 15 datasets containing primary and metastatic CRCs, including GSE6988 [17], GSE18105 [26], GSE21510 [18], GSE27854 [27], GSE28722 [28], GSE29623 [29], GSE38832 [30], GSE40967 [31], GSE41258 [22], GSE41568 [32], GSE62322 [33], GSE64258 [34], GSE71222 [34], GSE81582 [35], and GSE81986 [36], were retrieved from GEO. These 15 datasets include 1607 primary and 581 metastatic CRCs.

### 2.4 Ectopic expression or silencing of GABRD, and transfection

Lentiviral plasmids expressing GABRD (using GV144 vector), short hairpin RNA (shRNA) oligos of GABRD (using GV248 vector), or respective controls were constructed by Shanghai Genechem Co., Ltd (Shanghai, China). The target sequences were CAGACACCATTGACATTTA (shGABRD-1), CTCATTTCAACGCCGACTA (shGABRD-2), TGACGATGACCACGCTCAT (shGABRD-3), GTTACTCATCGGAGGACAT (shGABRD-4), and TTCTCCGAACGTGTCACGT (scramble control). Transient transfection of cells was performed using Lipofectamine 3000 from Invitrogen (L3000015, San Diego, CA, USA) according to the manufacturer’s instruction. To select stable transfectants, puromycin with a concentration of 0.4 µg/mL was used (abs42025969, Absin Bioscience Inc., Shanghai, China). The medium containing puromycin was changed every two days to remove dead cells. Once establishment of stable transfectants was confirmed, puromycin treatment was reduced to 0.2 µg/mL.

### 2.5 RNA extraction and the quantitative polymerase chain reaction (q-PCR)

Tissue and cellular RNA extraction and q-PCR were performed, as previously described [37]. The sequences for q-PCR primers were: GABRD forward primer, 5′-GCATCCGAATCACCTCCACTG-3′; and GABRD reverse primer, 5′-GATGAGTAACCGTAGCTCTCCA-3′. The specificity of primers was validated by electrophoresis and sequencing. Glyceraldehyde 3-phosphate dehydrogenase (GAPDH) was used as an internal reference. Experiments were performed three times in duplicate.

### 2.6 Western blotting

Total cellular protein extraction and western blotting were performed, as previously described [37]. The following antibodies were used: a rabbit anti-GABRD polyclonal antibody (1:1,000 dilution; abs141150; Absin Bioscience Inc., Shanghai, China) and a rabbit anti-GAPDH polyclonal antibody (1:2,000 dilution; Beyotime Biotechnology, Shanghai, China) as the loading control.

### 2.7 Cell proliferation assay

Stably transfected HCT15 and HCT116 cells (5 × 10^3^ cells/well) were seeded in 96-well plates and cultivated overnight. Then proliferation assays were performed, as previously described [37].

### 2.8 Scratch wound healing assay

Stably transfected HCT15 and HCT116 cells (4 × 10^5^ cells/well) were seeded in 12-well plates and cultivated until 100% confluence. Then monolayer scratch wound healing assays were performed, as previously described [37].

### 2.9 Statistical analysis

Paired or unpaired Student’s *t* tests were used for continuous variables. The Fisher exact test and chi-square tests were utilized for categorical comparisons. Survival analyses were evaluated by the Kaplan–Meier method. Univariate and multivariate survival analyses were conducted with the Cox proportional hazards regression model. All tests and reported *p* values were two-sided, and *p* < 0.05 was defined as statistically significant. Statistical analyses were performed with the Microsoft Excel 2010 (Microsoft, Redmond, WA, USA), GraphPad Prism7 (GraphPad, San Diego, CA, USA), and SPSS software version 22 for Windows (SPSS Inc., Chicago, Ill, USA).

## 3 Results

### 3.1 Expression of GABRD was increased in CRCs

**Figure 1:**
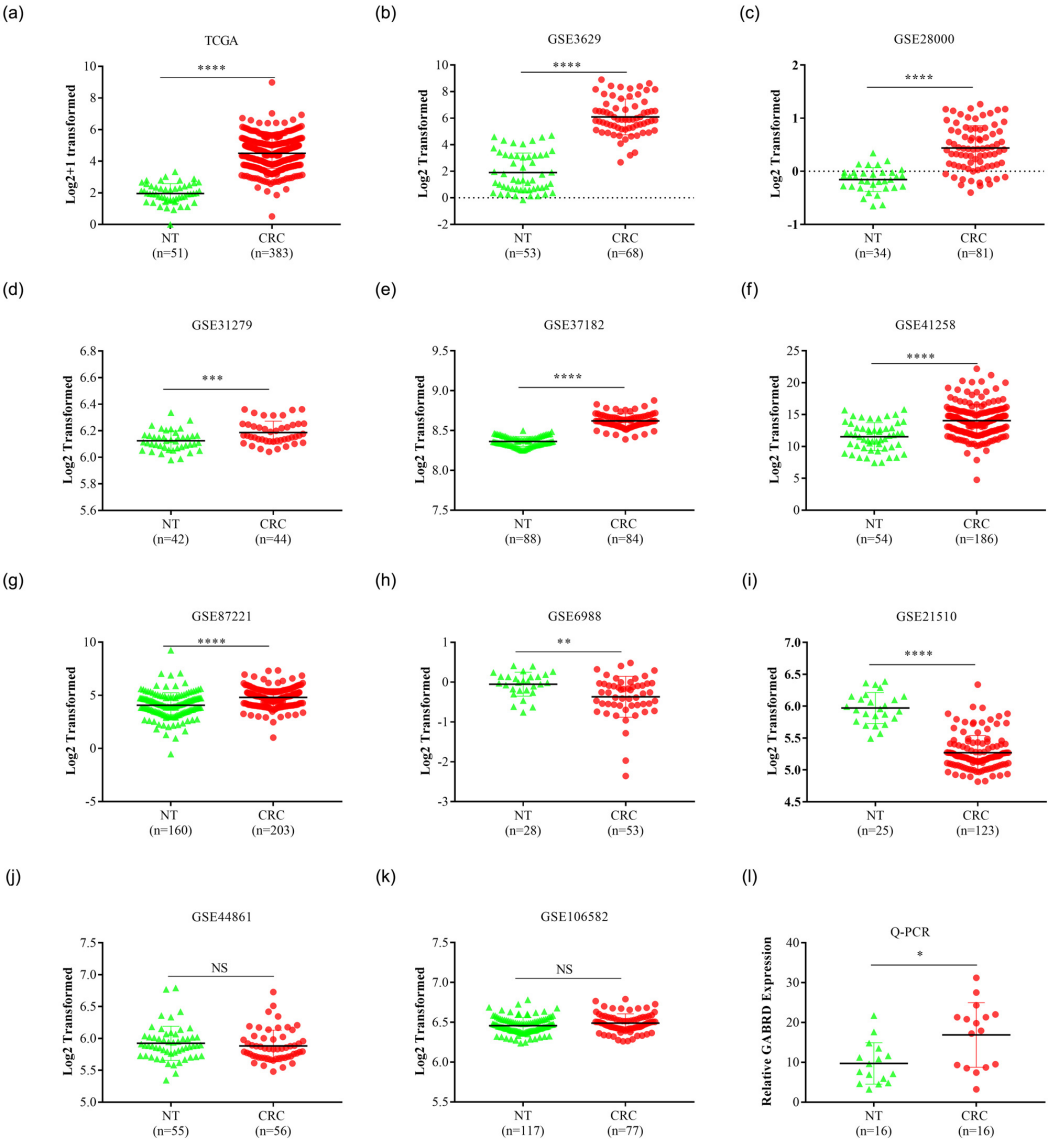
GABRD transcription was increased in CRCs. Expression of GABRD was compared between CRCs and adjacent NTs using transcriptomic data from one combined TCGA-COAD and READ dataset and 10 datasets in GEO. The results demonstrated that the GABRD expression was significantly increased in seven datasets (a–g), decreased in two datasets (h–i), and similar in two datasets (j–k) in CRCs compared to that in NTs. Sixteen paired CRCs and NTs from clinically resected samples were used to validate results from the transcriptomic datasets using q-PCR (l). Abbreviations: CRC, colorectal cancer; GABRD, gamma-aminobutyric acid type A receptor subunit delta; GEO, gene expression omnibus; NS, not significant; NT, adjacent normal tissue; q-PCR, quantitative polymerase chain reaction; TCGA-COAD and READ, the cancer genome atlas colon adenocarcinoma and rectal adenocarcinoma. *p < 0.05, **p < 0.01, ***p < 0.001, ****p < 0.0001.

As GABRD had not been investigated in CRCs previously, we first explored the expression pattern of GABRD in CRCs compared to that in NTs using 11 public transcriptomic datasets. As shown in Figure 1, GABRD was significantly upregulated in seven datasets (Figure 1a–g) and decreased in two datasets (Figure 1h–i), and was similar in two datasets (Figure 1j–k), in CRCs compared to that in NTs. We further compared the mRNA expression of GABRD between CRCs and NTs using clinically resected samples. The result confirmed that GABRD was increased in CRCs than in NTs (Figure 1l). We then wanted to make sure whether this was also the case in metastatic CRCs. In a panel of 15 transcriptomic datasets, no significant difference was observed with regard to GABRD expression in 14 of them in metastatic CRCs compared to that in primary CRCs (Figure S1a–n). While in one dataset that included different disease stages, GABRD expression was significantly increased in polyps and primary tumors compared to that in mucosae, and slightly but significantly decreased in metastatic CRCs compared to primary tumors, but was not significantly different between primary tumors and polyps (Figure 1o). Taken together, these results demonstrated that GABRD was upregulated in CRCs and might be involved in early tumorigenesis.

### 3.2 Overexpression of GABRD predicted unfavorable patient prognoses in CRC patients

**Figure 2:**
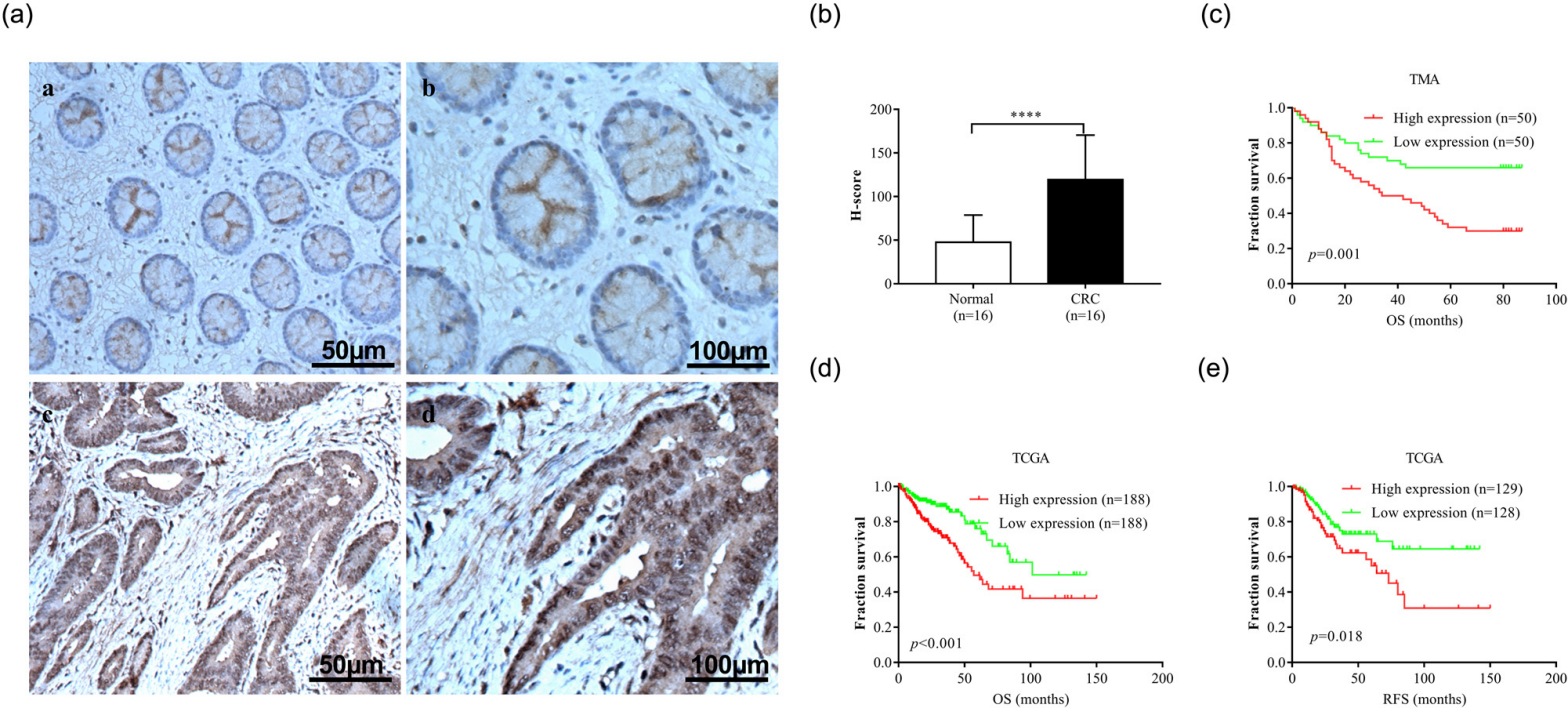
Overexpression of GABRD was associated with poor patient prognosis. The protein expression and localization of GABRD were characterized by IHC in 16 paired NTs and CRCs. GABRD expression was weak or negative in NTs and mainly localized to the cytoplasm and membrane (a, a and b). By contrast, GABRD expression was stronger and localized to the nuclei, cytoplasm, and membrane in CRCs (a, c and d; b). The prognostic value of GABRD in CRCs was explored with a TMA and validated using the combined TCGA-COAD and READ dataset. Kaplan–Meier plots demonstrated that the overexpression of GABRD was associated with unfavorable patient OS in the TMA cohort (c) and TCGA cohort (d), and unfavorable patient RFS in the TCGA cohort (e). *P* values were obtained by using the log-rank test. Censored data are indicated by the + symbol. Patients were stratified into GABRD low and high expression according to the median H score or GABRD mRNA expression (<median vs ≥median). Abbreviations: CRC, colorectal cancer; GABRD, gamma-aminobutyric acid type A receptor subunit delta; IHC, immunohistochemistry; NT, normal tissue; OS, overall survival; RFS, recurrence-free survival; TCGA-COAD and READ, the cancer genome atlas colon adenocarcinoma and rectal adenocarcinoma; TMA, tissue microarray. ****p < 0.0001.

We next determined the relationship between the overexpression of GABRD and patient outcome. We first performed IHC on 16 paired CRCs and NTs from clinically resected samples. As shown in Figure 2a, GABRD staining was weak or negative in NTs and localized to the cytoplasm and membrane (Figure 2a and b). By contrast, GABRD expression was stronger and localized to the nuclei, cytoplasm, and membrane in CRCs (Figure 2c and d), consistent with those observed from the Human Protein Atlas database (http://www.proteinatlas.org/ENSG00000187730-GABRD/pathology). An evaluation of the sections using H score revealed significant difference between CRCs and NTs with regard to GABRD staining (*p* < 0.000, Figure 2b). We then evaluated the correlation between GABRD expression and clinicopathological variables. As shown in Table 1, overexpression of GABRD was significantly correlated with later pTNM stages. We next determined the prognostic role of GABRD in CRC patients using a TMA. As shown in Figure 2c, a high expression of GABRD was associated with unfavorable overall survival (OS) in these patients (estimated mean OS 44.9 [95% CI, 36.2–53.7] months vs 63.7 [95% CI, 54.5–73.0] months, log-rank *p* = 0.001). In the multivariate analysis using a Cox proportional hazards model, GABRD overexpression was significantly and independently associated with shorter OS, after adjustment by age, tumor size, and tumor stage (Table 2). Besides, GABRD overexpression was significantly associated with shorter OS and recurrence-free survival (RFS) in the combined TCGA-COAD and READ cohort, which supported the observation in the TMA cohort (Figure 2d and e). Collectively, these results demonstrated that the overexpression of GABRD was predictive of unfavorable prognoses in CRC patients.

**Table 1:** Clinical significance of the GABRD expression in colorectal cancers (*n* = 100)

Clinicopathological features	Number of patients	GABRD expression		
		Low (50)	High (50)	*p*-value
**Sex**				
Male	59	28 (47.5)	31 (52.5)	0.342
Female	41	22 (53.7)	19 (46.3)	
**Age**				
<69	50	29 (58)	21 (42)	0.159
≥69	50	21 (42)	29 (58)	
**Histological grade**				
G2	70	35 (50)	35 (50)	0.586
G3	30	15 (50)	15 (50)	
**Tumor size (cm)**				
<5	36	17 (47.2)	19 (52.8)	0.447
≥5	63	32 (50.8)	31 (49.2)	
**pT stage**				
T2/T3	68	32 (47.1)	36 (52.9)	0.260
T4	32	18 (56.3)	14 (43.7)	
**pN stage**				
N0	52	31 (59.6)	21 (40.4)	0.036
N1/N2	48	19 (39.6)	29 (60.4)	
**p stage**				
I/II	51	31 (60.8)	20 (39.2)	0.022
III/IV	49	19 (38.8)	30 (61.2)	

Abbreviations: GABRD, gamma-aminobutyric acid type A receptor subunit delta.

**Table 2:** Univariate and multivariate models for overall survival in the TMA cohort (*n* = 100)

Clinicopathological features	Univariate analysis		Multivariate analysis	
	HR [95% CIs]	*p*-value	HR [95% CIs]	*p*-value
**Sex**				
Male	1 [reference]			
Female	1.34 [0.76–2.35]	0.315		
**Age**				
<69	1 [reference]		1 [reference]	
≥69	0.54 [0.31–0.95]	0.032	0.47 [0.26–0.85]	0.013
**Histological grade**				
G2	1 [reference]			
G3	0.74 [0.42–1.32]	0.310		
**Tumor size (cm)**				
<5	1 [reference]		1 [reference]	
≥5	0.58 [0.34–1.01]	0.053	0.46 [0.26–0.82]	0.009
**pT stage**				
T1/T2	1 [reference]			
T3/T4	0.84 [0.47–1.48]	0.538		
**pN stage**				
N0	1 [reference]			
N1–N3	0.62 [0.36–1.06]	0.082		
**pStage**				
I/II	1 [reference]		1 [reference]	
III/IV	0.58 [0.33–1.01]	0.052	0.45 [0.24–0.83]	0.011
**GABRD expression**				
Low	1 [reference]		1 [reference]	
High	0.40 [0.22–0.71]	0.002	0.51 [0.28–0.95]	0.033

Abbreviations: TMA, tissue microarray; HR, hazard ratio; CI, confidence interval; GABRD, gamma-aminobutyric acid type A receptor subunit delta.

### 3.3 Overexpression of GABRD promoted the proliferation and migration of CRC cells

**Figure 3:**
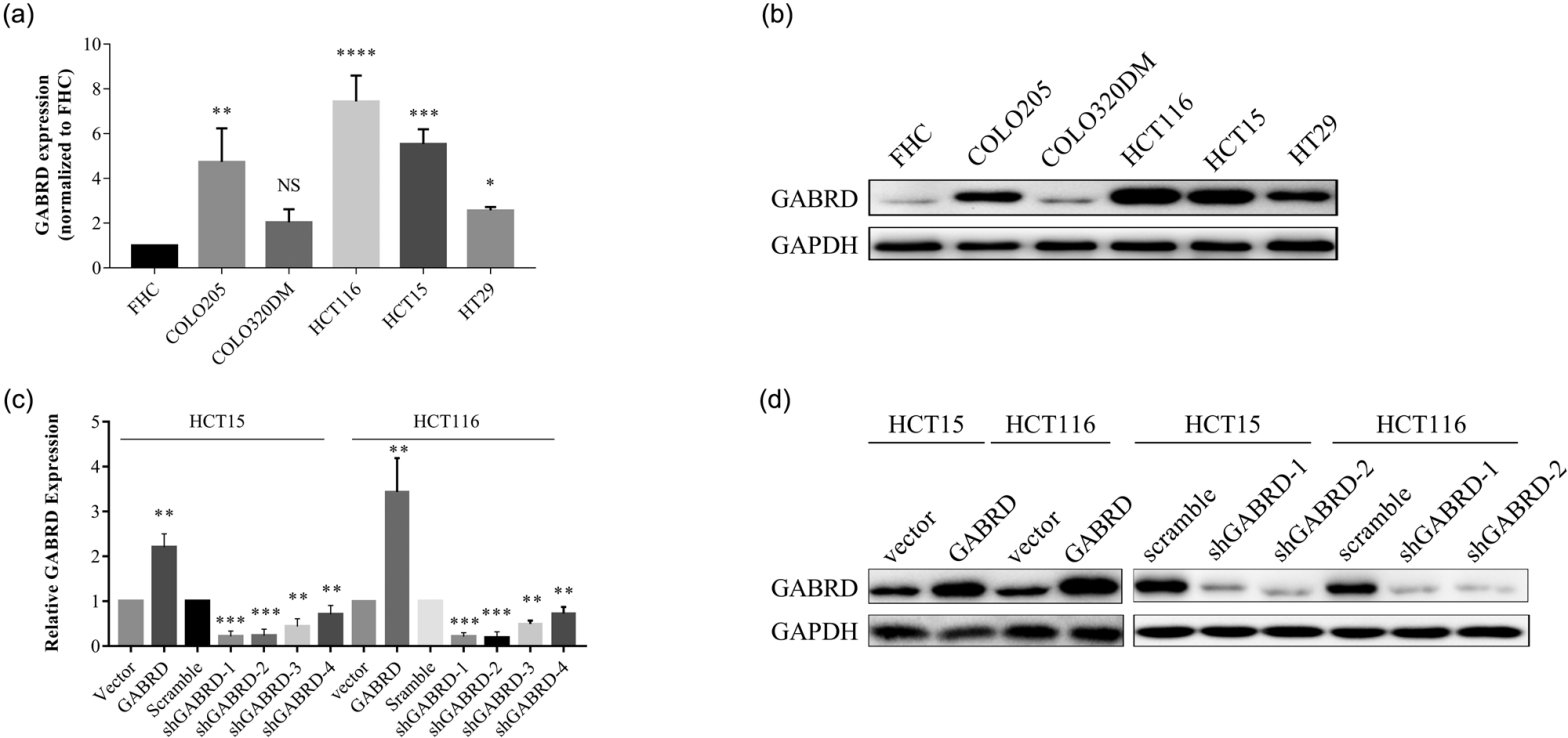
GABRD expression was upregulated in cultured CRC cell lines. The mRNA and protein expression of GABRD was investigated in a colon epithelial cell line FHC and five CRC cell lines using q-PCR and western blotting. The results demonstrated that the GABRD expression was dramatically increased in four of the CRC cell lines compared to that in the colon epithelial cell line FHC (a and b). Two CRC cell lines were selected and transduced with vector, GABRD, scramble shRNA, or GABRD shRNAs, then underwent q-PCR and western blotting to detect GABRD expression in these cells (c and d). Abbreviations: CRC, colorectal cancer; GABRD, gamma-aminobutyric acid type A receptor subunit delta; GAPDH, glyceraldehyde 3-phosphate dehydrogenase; NS, not significant; q-PCR, quantitative polymerase chain reaction. *p < 0.05, **p < 0.01, ***p < 0.001, ****p < 0.0001.

**Figure 4:**
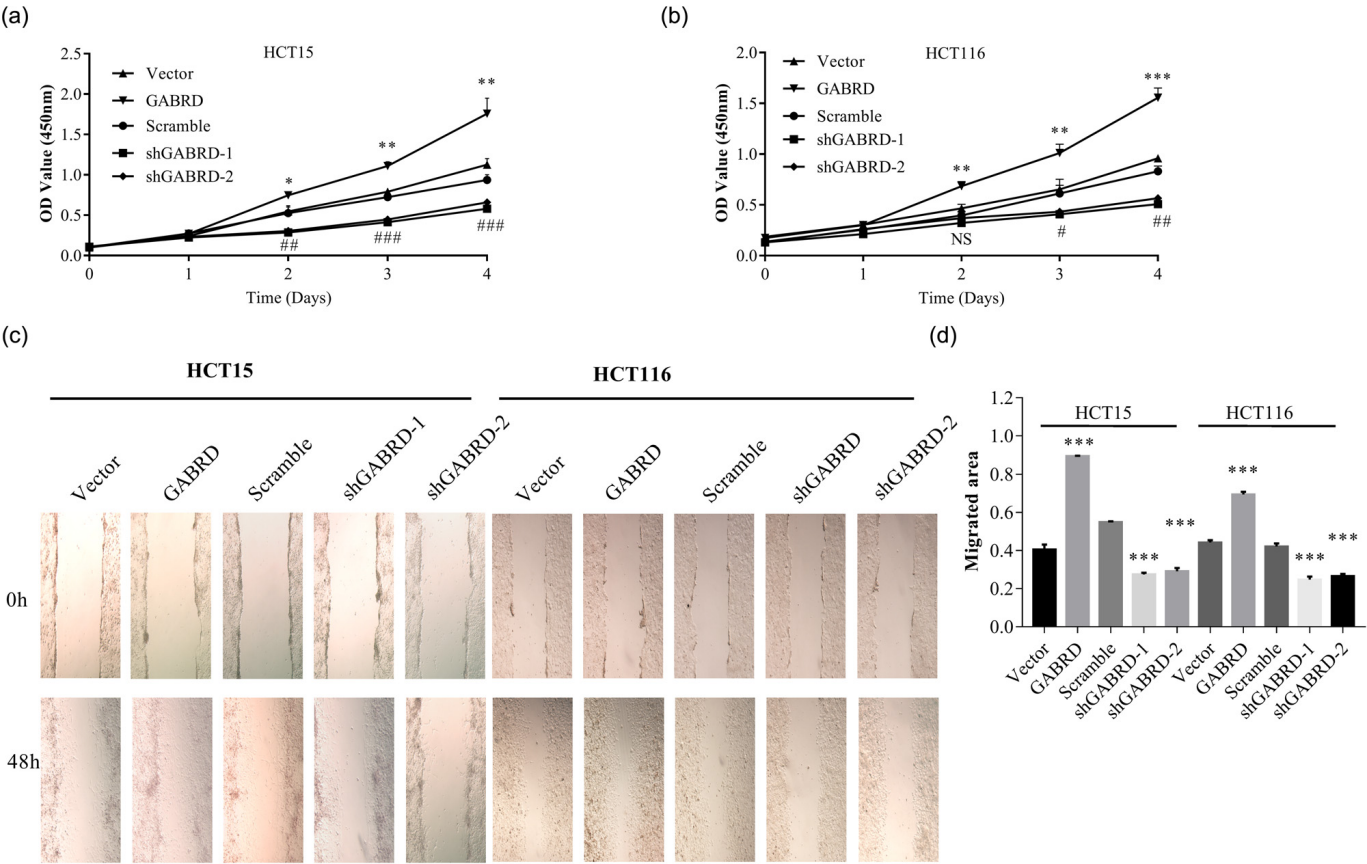
GABRD promoted CRC cell proliferation and migration. HCT15 and HCT116 cells were transduced with vector, GABRD, scramble shRNA, or shGABRDs, and underwent CCK-8 cell proliferation assay and monolayer wound healing assay. Overexpression of GABRD promoted while knock-down of GABRD impeded cell proliferation (a and b) and migration (c and d) of these cells. Abbreviations: CCK-8, cell counting kit-8; CRC, colorectal cancer; GABRD, gamma-aminobutyric acid type A receptor subunit delta; NS, not significant. *p < 0.05, **p < 0.01, ***p < 0.001; #, ##, and ### represent *p* < 0.05, *p* < 0.01, and *p* < 0.001, respectively, for comparisons between scramble and shGABRD-transduced cells in (a and b). Data representative of three replicated experiments are given.

To further examine the role of GABRD in CRCs, we first examined the endogenous expression of GABRD mRNA and protein in a colon epithelial cell line FHC and five CRC cell lines using q-PCR and western blotting. The results demonstrated that the GABRD expression was dramatically increased in four of the CRC cell lines compared to that in FHC cells (Figure 3a and b). Two CRC cell lines were selected and transduced with vector, GABRD, scramble shRNA, or GABRD shRNAs, then underwent q-PCR and western blotting to detect the GABRD expression in these cells (Figure 3c and d). We next investigated the in vitro activities of GABRD using CCK-8 cell proliferation assay and monolayer scratch wound healing assay. As shown in Figure 4, overexpression of GABRD promoted while knock-down of GABRD impeded cell proliferation (Figure 4a and b) and migration (Figure 4c and d).

**Figure 5:**
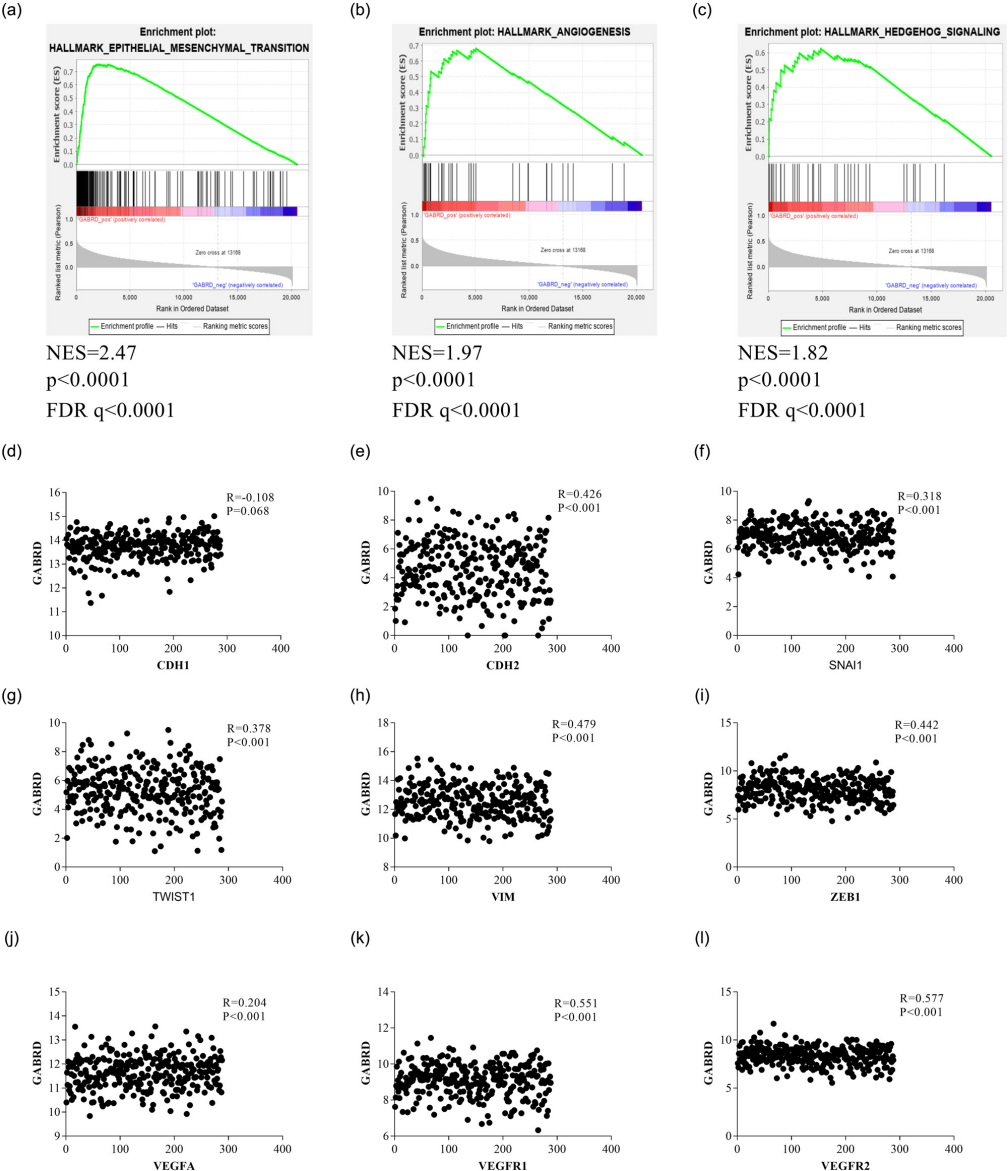
Potential mechanisms underlying the role of GABRD in CRCs. A GSEA was performed using the TCGA-COAD dataset to explore possible mechanisms underlying GABRD’s role in carcinogenesis and progression of CRCs. The results indicated that a high expression of GABRD was positively correlated with hallmark gene sets defining epithelial–mesenchymal transition, angiogenesis, and hedgehog signaling. Gene–gene correlation analyses demonstrated that GABRD was significantly correlated with several key genes associated with EMT and angiogenesis. Abbreviations: CDH1, cadherin 1; CDH2, cadherin 2; CRC, colorectal cancer; FDR, false discovery rate; GABRD, gamma-aminobutyric acid type A receptor subunit delta; GSEA, gene sets enrichment analysis; NES, normalized enrichment score; SNAI1, snail family transcriptional repressor 1; TCGA-COAD, the cancer genome atlas colon adenocarcinoma; TWIST1, twist family BHLH transcription factor 1; VEGFA, vascular endothelial growth factor A; VEGFR, vascular endothelial growth factor receptor; VIM, vimentin; ZEB1, zinc finger E-box binding homeobox 1.

We next wanted to explore the possible mechanisms underlying GABRD’s role in progression of CRCs. A GSEA was performed using the TCGA-COAD dataset. As shown in Figure 5, a high expression of GABRD was positively correlated with hallmark gene sets defining epithelial–mesenchymal transition (EMT), angiogenesis, and hedgehog signaling (Figure 5a–c), as well as KRAS signaling, Wnt-β-catenin signaling, UV response, etc. (data not shown). We then conducted gene–gene correlation analyses using the same dataset. Figure 5d–l demonstrates that the expression of GABRD was correlated with those of EMT markers (except that of CDH1/E-cadherin) and VEGFA and its two receptors, but not with those of the key markers of the hedgehog signaling pathway (data not shown).

Taken together, these observations suggested that GABRD promoted CRC progression by enhancing cell proliferation and migration, and interacting with crucial pathways involved in tumor progression.

## 4 Discussion

In the current study, we found that the expression of GABRD was significantly increased in CRCs compared to that in NTs, but not in metastasis than that in primary tumors. Besides, overexpression of GABRD in CRCs was associated with later TNM stages and shorter survival times. Overexpression of GABRD accelerated tumor cell proliferation and migration, while silencing of GABRD yielded the opposite effects. Finally, a high expression of GABRD was positively correlated with hallmark gene sets defining epithelial–mesenchymal transition, angiogenesis, and hedgehog signaling.

Research works of the GABA_A_ receptors predominantly focus on the central nervous system [5]. GABRD, which encodes one of the GABA_A_ receptors, has been related to some nervous disorders [7,8]. Although Gross et al. observed a ubiquitous overexpression of GABRD across various cancer types in the TCGA dataset, these authors did not go any further to explore the prognostic significance of GABRD overexpression in specific tumor types [10]. GABRD has also been referred to in tumors in several other studies [9,11,38,39], but many of these are based on bioinformatic data and the functional relevance of GABRD to carcinogenesis and tumor progression remains to be elucidated.

Using both transcriptomic data from several public datasets and IHC of clinically resected samples, we demonstrated that the GABRD expression was upregulated in CRCs compared to that in neighboring NTs, but similar between primary and metastatic CRCs. Besides, in a single dataset with samples from different disease stages, the expression of GABRD was significantly increased in polyps and CRCs compared to that in NTs, but was similar between polyps and CRCs. These observations suggested that GABRD may be involved in early tumorigenesis of CRC. It is well known that the carcinogenesis of CRC follows the typical polyps/adenoma-cancer model, which involves the dysregulation of multiple protection mechanisms [40,41,42]. Blocking this axis at earlier stages can help in a prophylactic sense. Therefore, GABRD may be a possible target for the prevention of CRC carcinogenesis.

In the current study, using a tissue microarray and IHC, we found that overexpression of GABRD was correlated with an unfavorable patient survival, which was confirmed by analyzing data from the combined TCGA-COAD and READ cohort. Because of the heterogeneity among patients, screening for potential prognostic predictors would help select patients at a higher risk to prescribe more personalized treatment and follow-up plans [41,43,44]. Although results from the present study still need validations, the prognostic value of GABRD is definitely worth further investigation.

GABRD was extensively localized in the cell nuclei of CRCs, but was barely seen in those of NTs, as indicated by IHC in the current study and that from the HPA database, suggesting that GABRD might be associated with malignant proliferation. *In vitro* evidence using both gain-of-function and loss-of-function experiments confirmed this functional relevance of GABRD in CRCs.

In the GSEA, a high expression of GABRD was positively correlated to several gene sets that are closely related to carcinogenesis and tumor progression, including EMT, angiogenesis, hedgehog signaling, KRAS signaling, Wnt-β-catenin signaling, and UV response. While EMT and angiogenesis are typically involved in tumor progression, dysregulation of the hedgehog signaling, KRAS signaling, Wnt-β-catenin signaling, and UV response could drive colorectal tumorigenesis [42,45,46,47,48]. Using gene–gene correlation analyses, we found that the expression of GABRD was significantly correlated with those of EMT markers (except that of CDH1/E-cadherin) and VEGFA and its two receptors, suggesting that EMT and angiogenesis could be the future directions to fully understand the mechanism of GABRD in CRC carcinogenesis.

Although the current study uncovered a novel role of GABRD in carcinogenesis of CRC, it has some intrinsic limitations which should be addressed in our future studies. First, it did not provide evidence to support the oncogenic roles of GABRD *in vivo*, which might be quite different considering the complex interactions between tumor and the microenvironment. Second, it only partly elucidated the potential molecule mechanism underlying GABRD’s activity. Third, as clinical evidence suggested that GABRD might have influence more on tumor carcinogenesis than on metastasis, while *in vitro* experiments indicated that it promoted tumor proliferation and migration. This functional predisposition needs to be proved by *in vivo* experiments and explained by mechanistic studies.

## 5 Conclusion

In summary, the current study presents a novel finding that GABRD is aberrantly expressed in CRCs and predictive of unfavorable prognosis. As *in vitro* experiments further confirm its involvement in tumor proliferation, we postulate that GABRD could be a novel prognostic predictor for CRC that deserves further investigation.
